# Supplementary figures and images for: Z-REX uncovers a bifurcation in function of Keap1 paralogs
Source: eLife. 2022 Oct 27;11:e83373. doi: 10.7554/eLife.83373 (PMC9754640; doi:10.7554/eLife.83373)

Figure 1—figure supplement 1

D

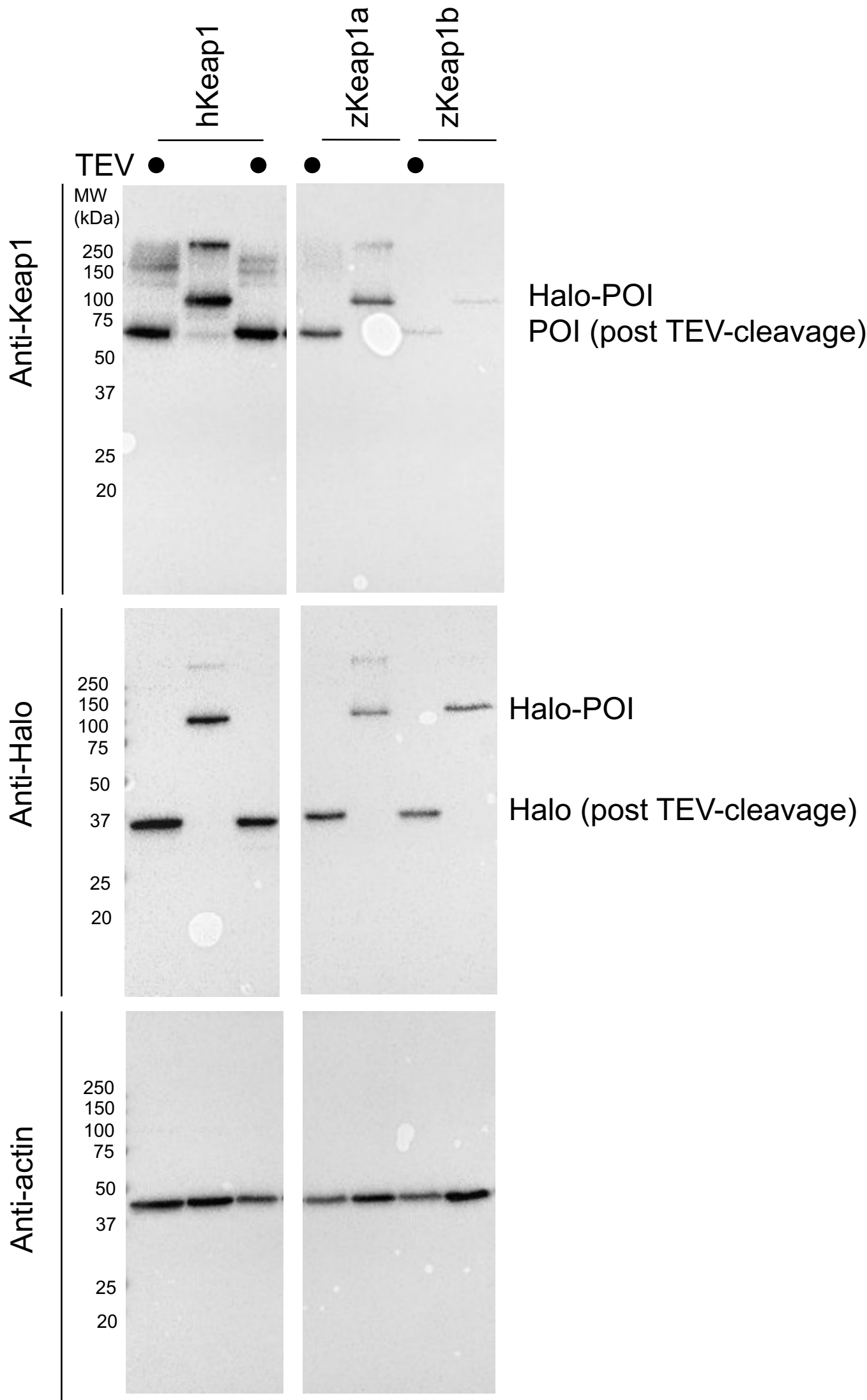

Supplement: Figure 1—figure supplement 1—source data 1. [file elife-83373-fig1-figsupp1-data1.zip › Figure 1-figure supplement 1-source data 1-full view blot image/full view blot.pdf]

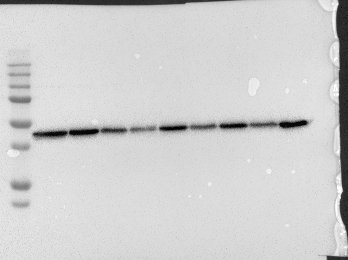

Supplement: Figure 1—figure supplement 1—source data 2. [file elife-83373-fig1-figsupp1-data2.zip › Figure 1-figure supplement 1-source data 2-raw blot images/Figure 1-figure supplement 1d-anti-actin.jpg]

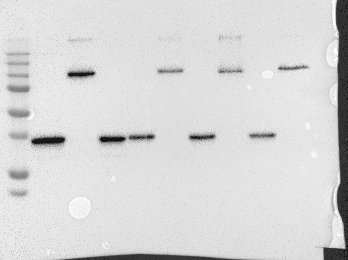

Supplement: Figure 1—figure supplement 1—source data 2. [file elife-83373-fig1-figsupp1-data2.zip › Figure 1-figure supplement 1-source data 2-raw blot images/Figure 1-figure supplement 1d-anti-Halo.jpg]

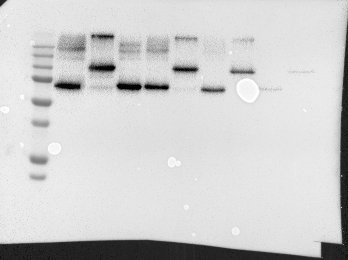

Supplement: Figure 1—figure supplement 1—source data 2. [file elife-83373-fig1-figsupp1-data2.zip › Figure 1-figure supplement 1-source data 2-raw blot images/Figure 1-figure supplement 1d-anti-Keap1.jpg]

Figure 1—figure supplement 4

A

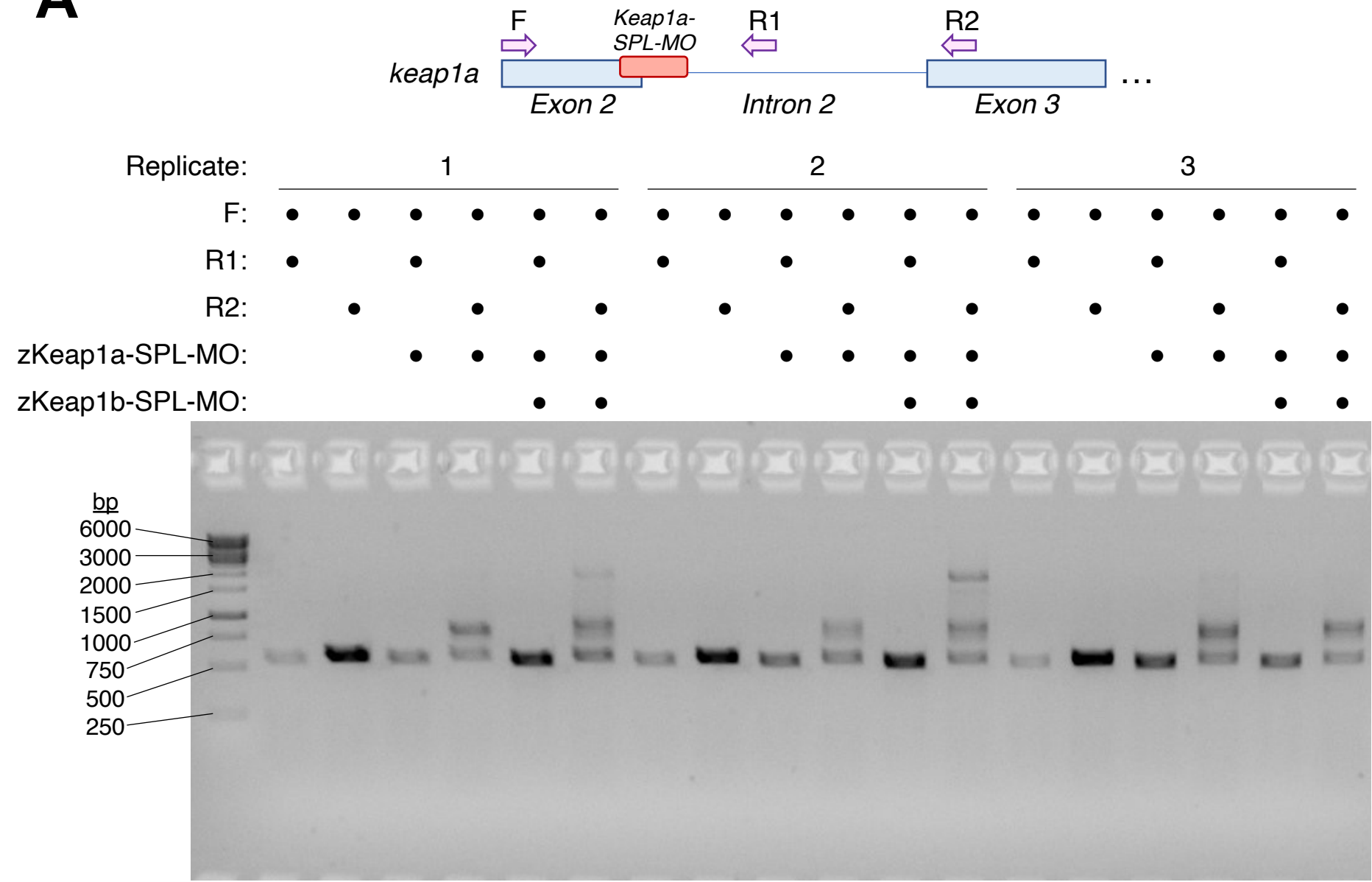

B

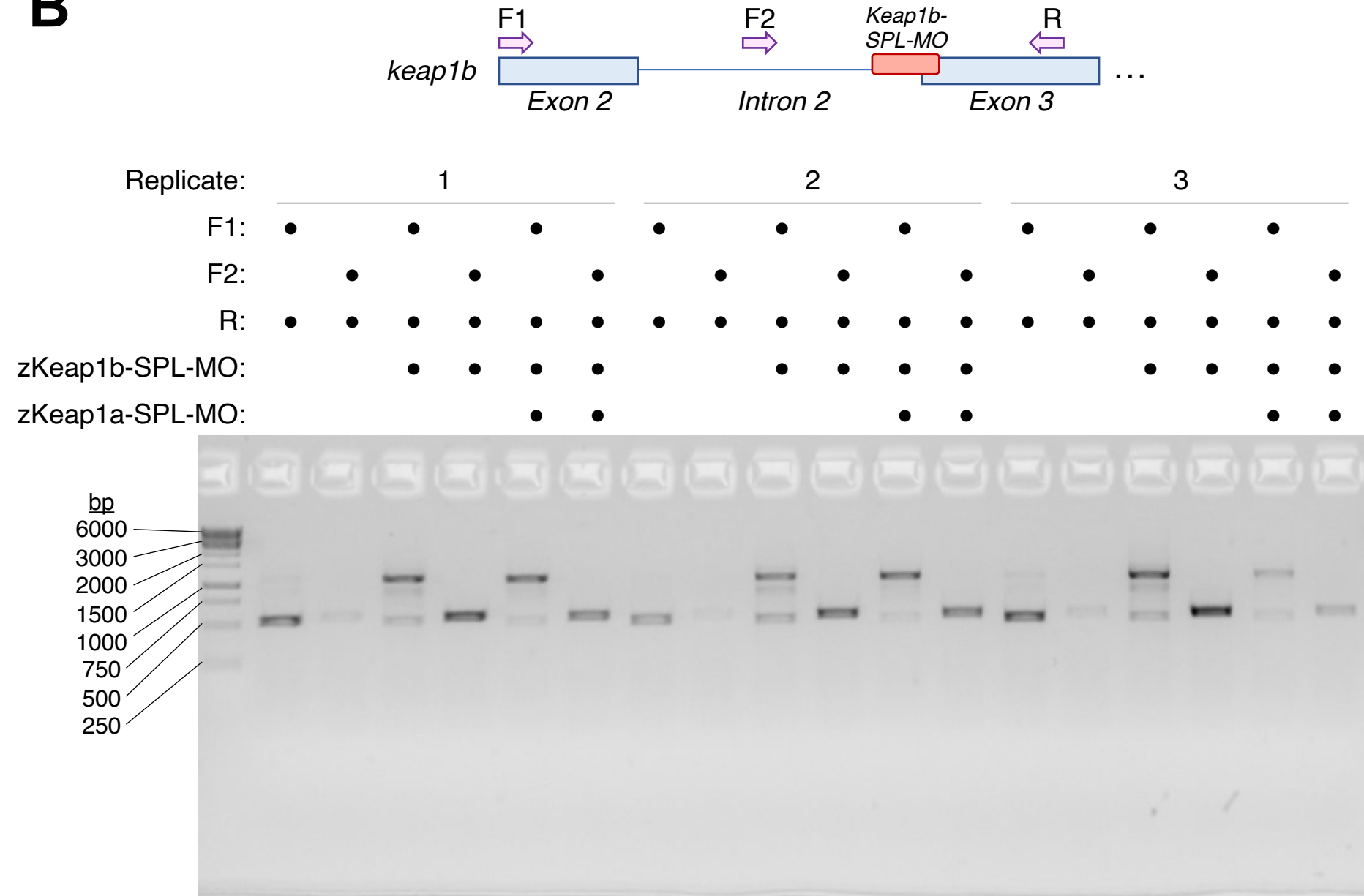

Supplement: Figure 1—figure supplement 4—source data 1. [file elife-83373-fig1-figsupp4-data1.zip › Figure 1-figure supplement 4-source data 1-full view gel image/full view gel.pdf]

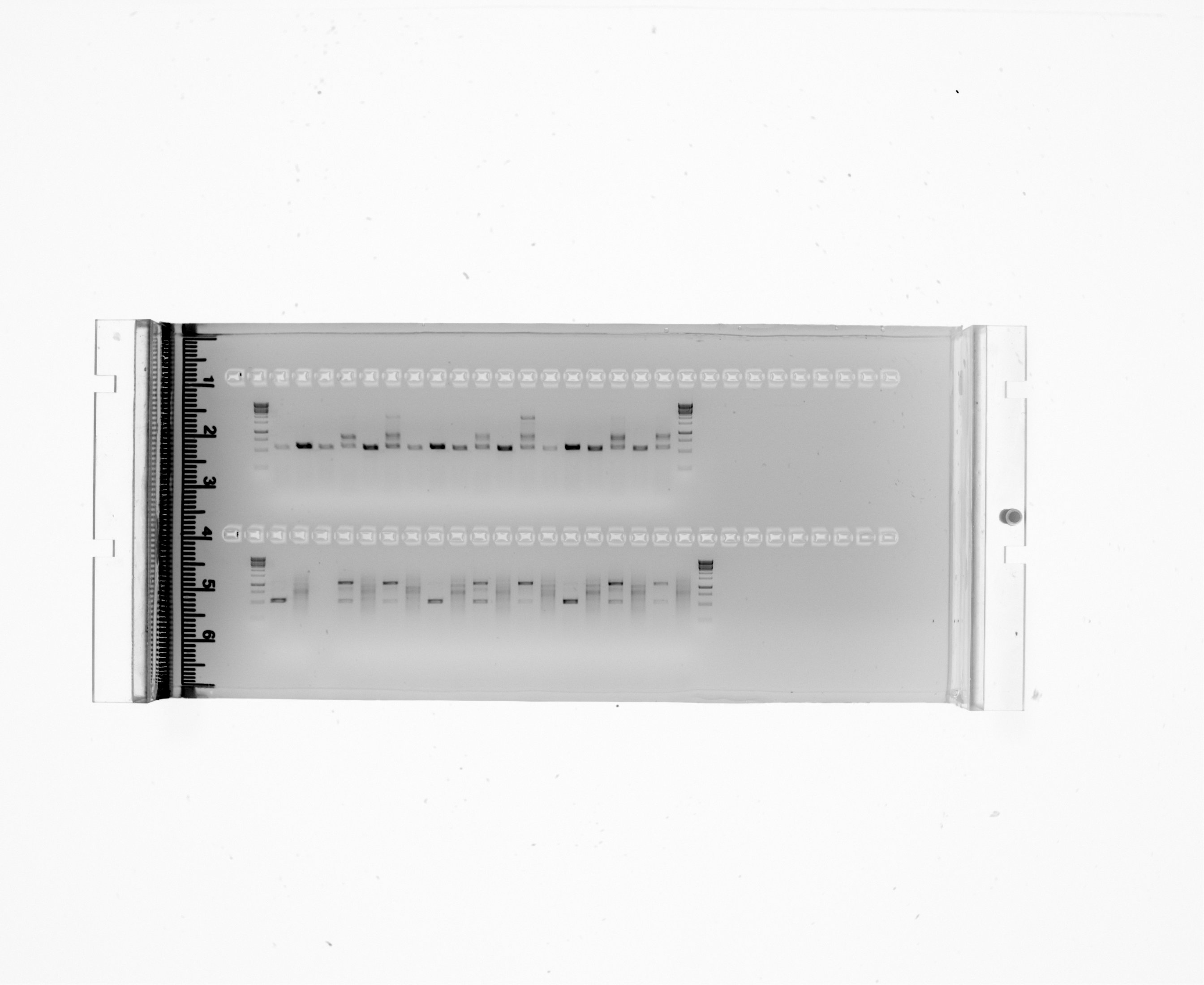

Supplement: Figure 1—figure supplement 4—source data 2. [file elife-83373-fig1-figsupp4-data2.zip › Figure 1-figure supplement 4-source data 2-raw gel image/Figure 1-figure supplement 4a.jpg]

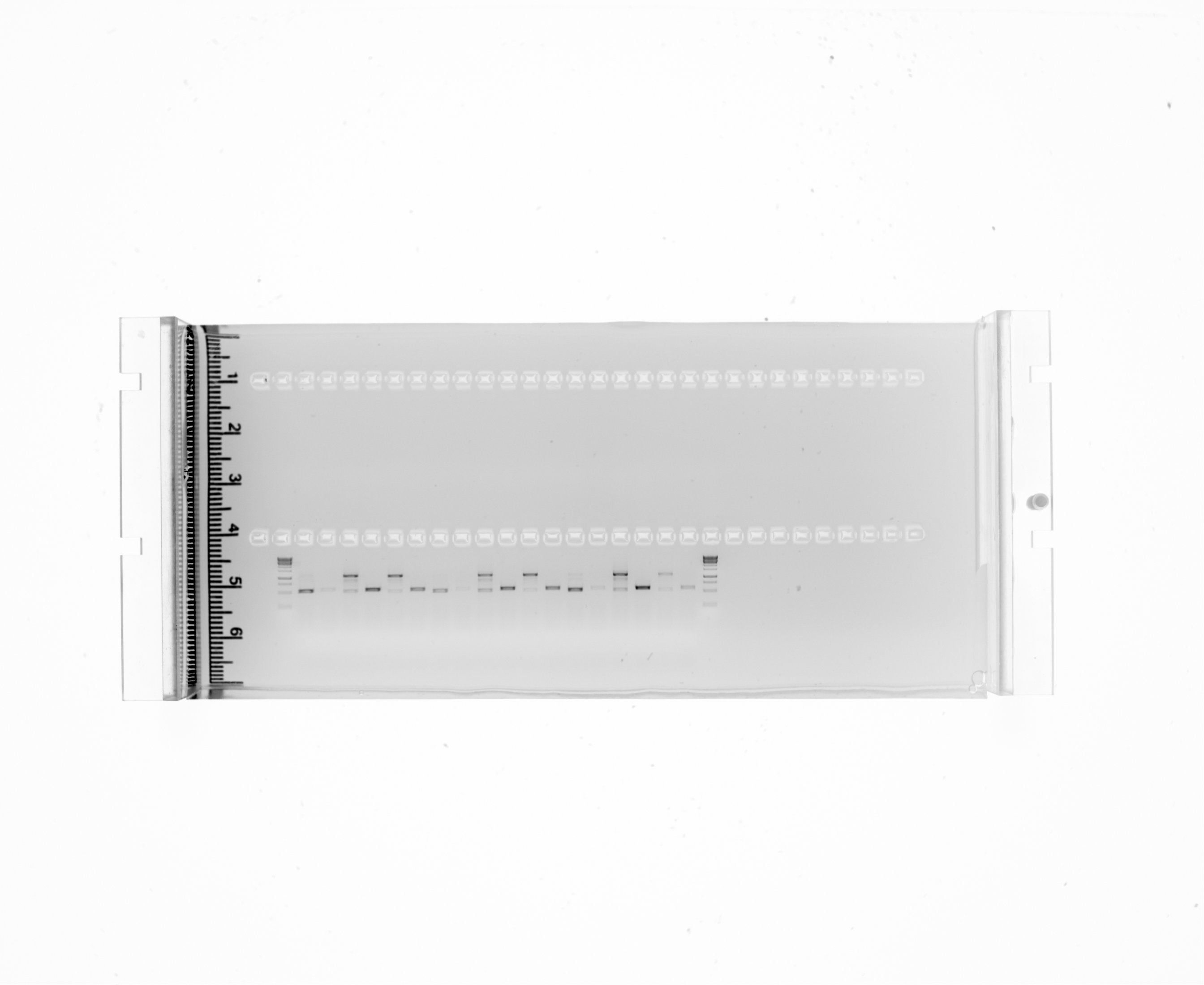

Supplement: Figure 1—figure supplement 4—source data 2. [file elife-83373-fig1-figsupp4-data2.zip › Figure 1-figure supplement 4-source data 2-raw gel image/Figure 1-figure supplement 4b.jpg]

Figure 3—figure supplement 3

B

Casper fish, Click-biotin pulldown

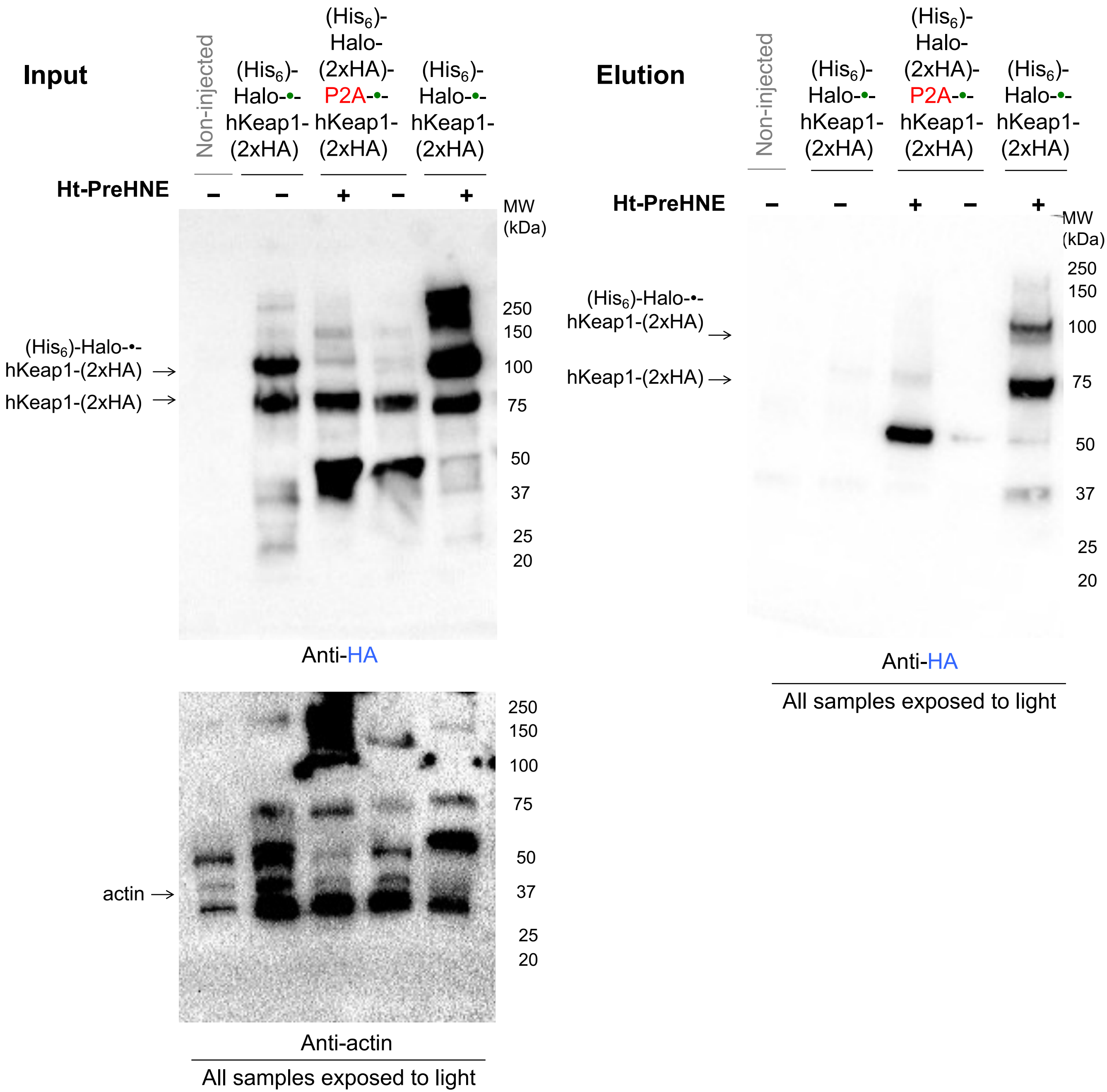

Supplement: Figure 3—figure supplement 3—source data 1. [file elife-83373-fig3-figsupp3-data1.zip › Figure 3-figure supplement 3-source data 1-full view blot image/full view blot.pdf]

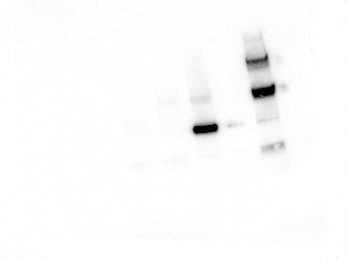

Supplement: Figure 3—figure supplement 3—source data 2. [file elife-83373-fig3-figsupp3-data2.zip › Figure 3-figure supplement 3-source data 2-raw blot image/Figure 3-figure supplement 3b-elution-anti-HA.jpg]

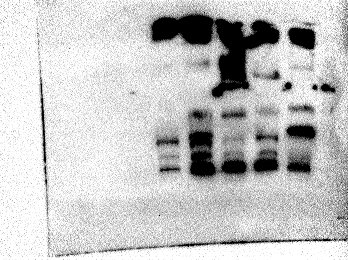

Supplement: Figure 3—figure supplement 3—source data 2. [file elife-83373-fig3-figsupp3-data2.zip › Figure 3-figure supplement 3-source data 2-raw blot image/Figure 3-figure supplement 3b-input-anti-actin.jpg]

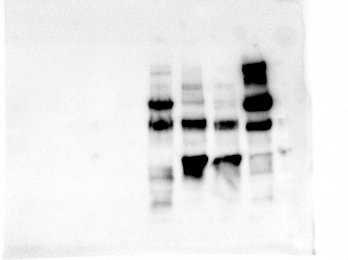

Supplement: Figure 3—figure supplement 3—source data 2. [file elife-83373-fig3-figsupp3-data2.zip › Figure 3-figure supplement 3-source data 2-raw blot image/Figure 3-figure supplement 3b-input-anti-HA.jpg]

Figure 3—figure supplement 4

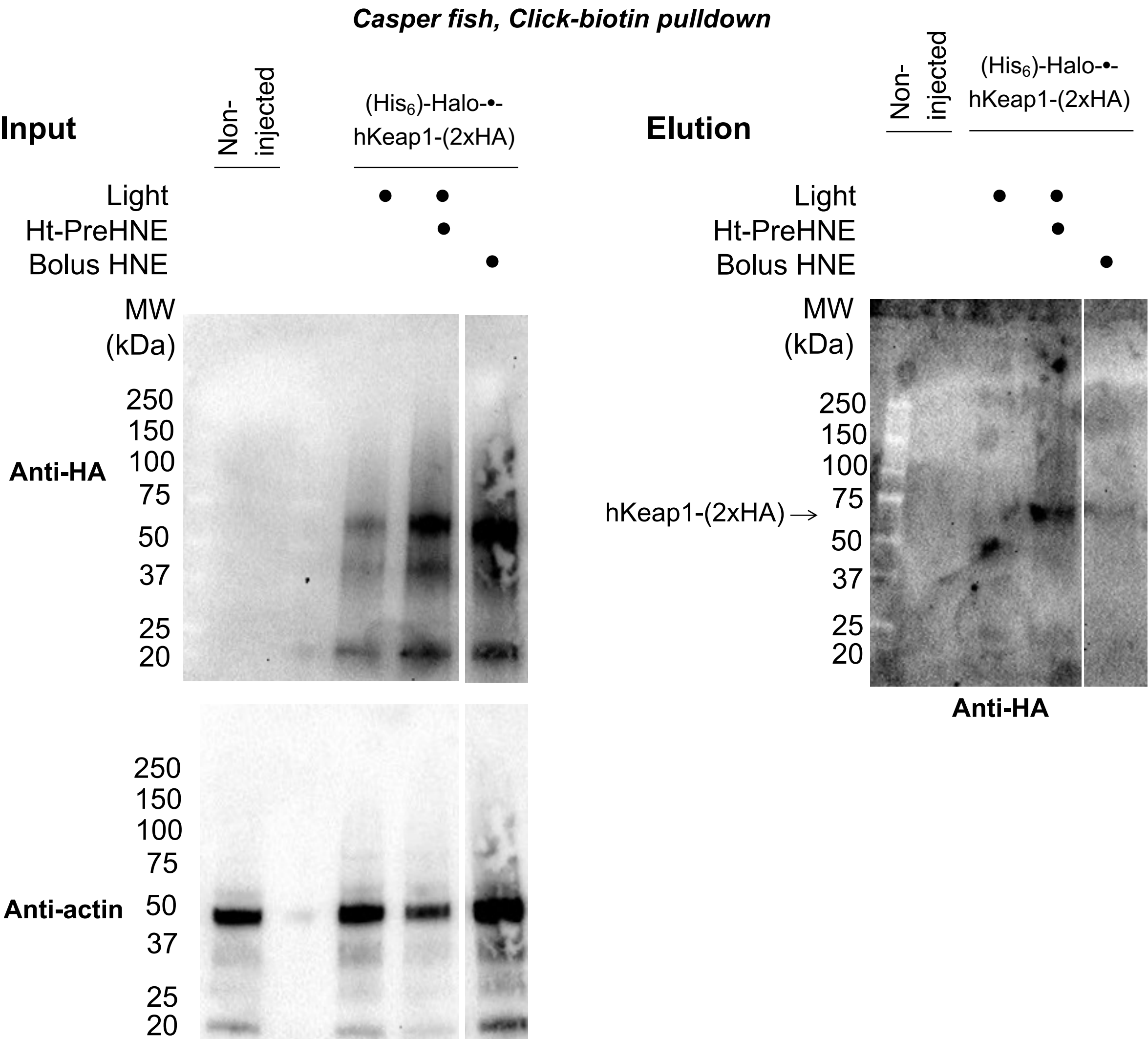

Supplement: Figure 3—figure supplement 4—source data 1. [file elife-83373-fig3-figsupp4-data1.zip › Figure 3-figure supplement 4-source data 1-full view blot image/full view blot.pdf]

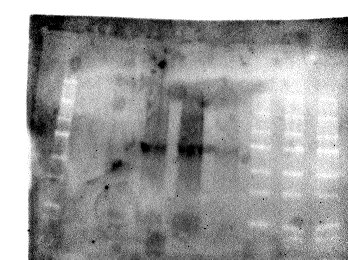

Supplement: Figure 3—figure supplement 4—source data 2. [file elife-83373-fig3-figsupp4-data2.zip › Figure 3-figure supplement 4-source data 2-raw blot image/Figure 3-figure supplement 4-elution-anti-HA.jpg]

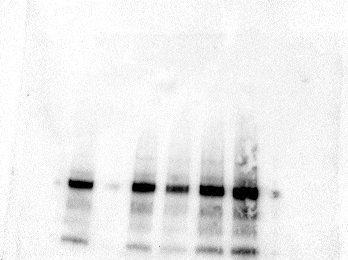

Supplement: Figure 3—figure supplement 4—source data 2. [file elife-83373-fig3-figsupp4-data2.zip › Figure 3-figure supplement 4-source data 2-raw blot image/Figure 3-figure supplement 4-input-anti-actin.jpg]

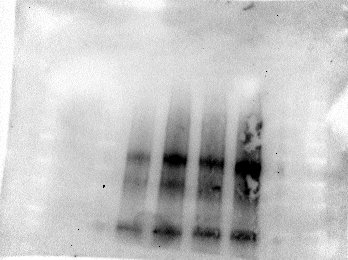

Supplement: Figure 3—figure supplement 4—source data 2. [file elife-83373-fig3-figsupp4-data2.zip › Figure 3-figure supplement 4-source data 2-raw blot image/Figure 3-figure supplement 4-input-anti-HA.jpg]

Figure 4—figure supplement 1

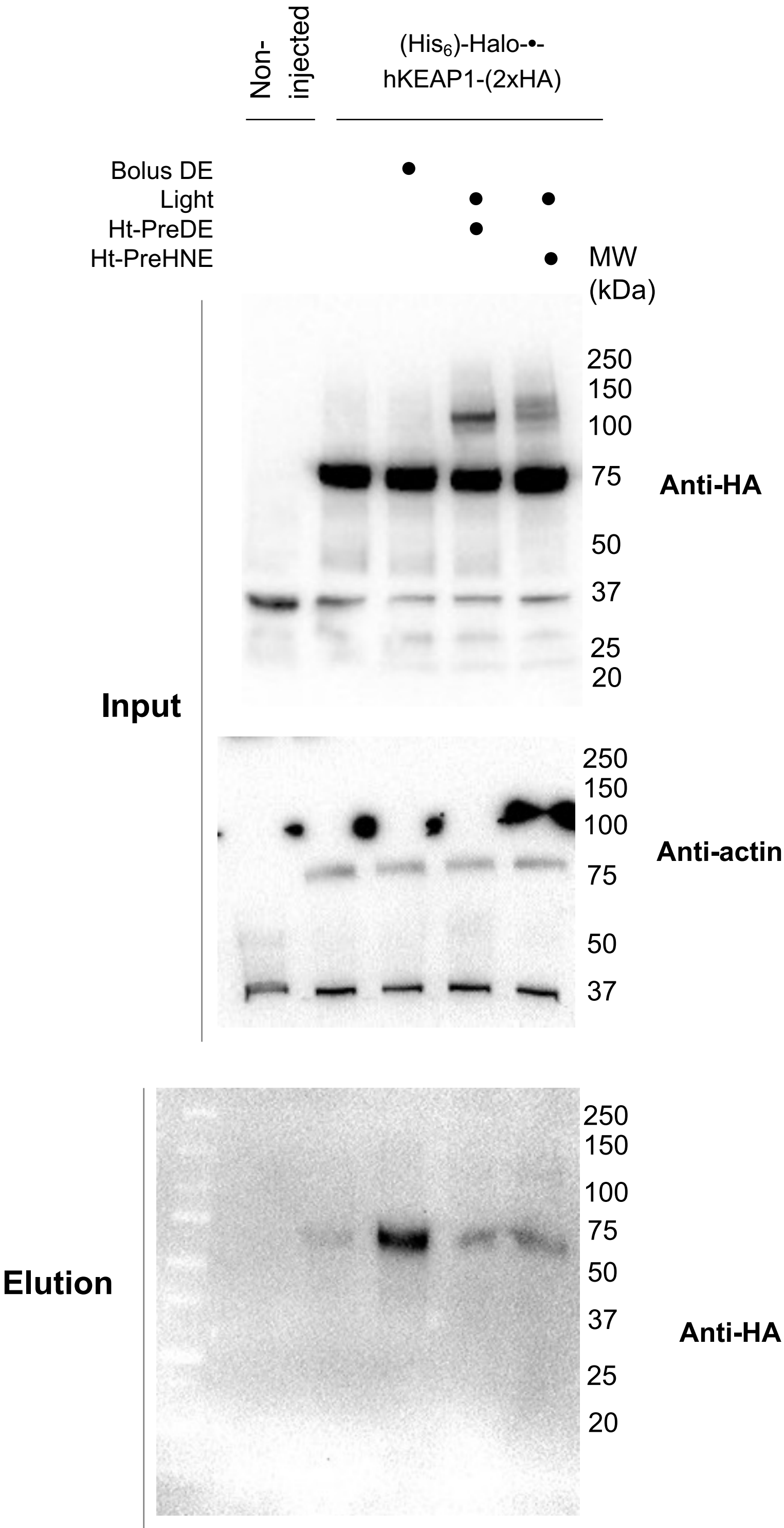

Supplement: Figure 4—figure supplement 1—source data 1. [file elife-83373-fig4-figsupp1-data1.zip › Figure 4-figure supplement 1-source data 1-full view blot image/full view blot.pdf]

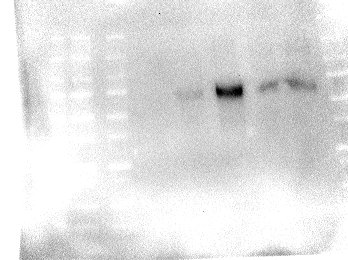

Supplement: Figure 4—figure supplement 1—source data 2. [file elife-83373-fig4-figsupp1-data2.zip › Figure 4-figure supplement 1-source data 2-raw blot image/Figure 4-figure supplement 1-elution-anti-HA.jpg]

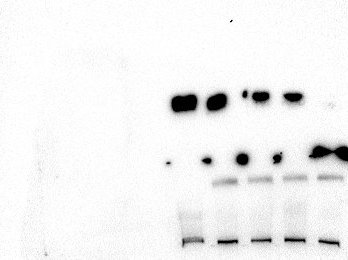

Supplement: Figure 4—figure supplement 1—source data 2. [file elife-83373-fig4-figsupp1-data2.zip › Figure 4-figure supplement 1-source data 2-raw blot image/Figure 4-figure supplement 1-input-anti-actin.jpg]

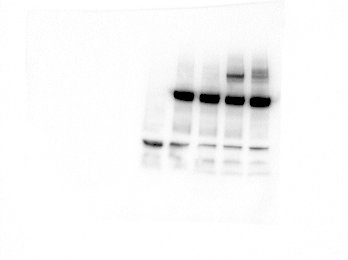

Supplement: Figure 4—figure supplement 1—source data 2. [file elife-83373-fig4-figsupp1-data2.zip › Figure 4-figure supplement 1-source data 2-raw blot image/Figure 4-figure supplement 1-input-anti-HA.jpg]

Figure 7—figure supplement 2

B

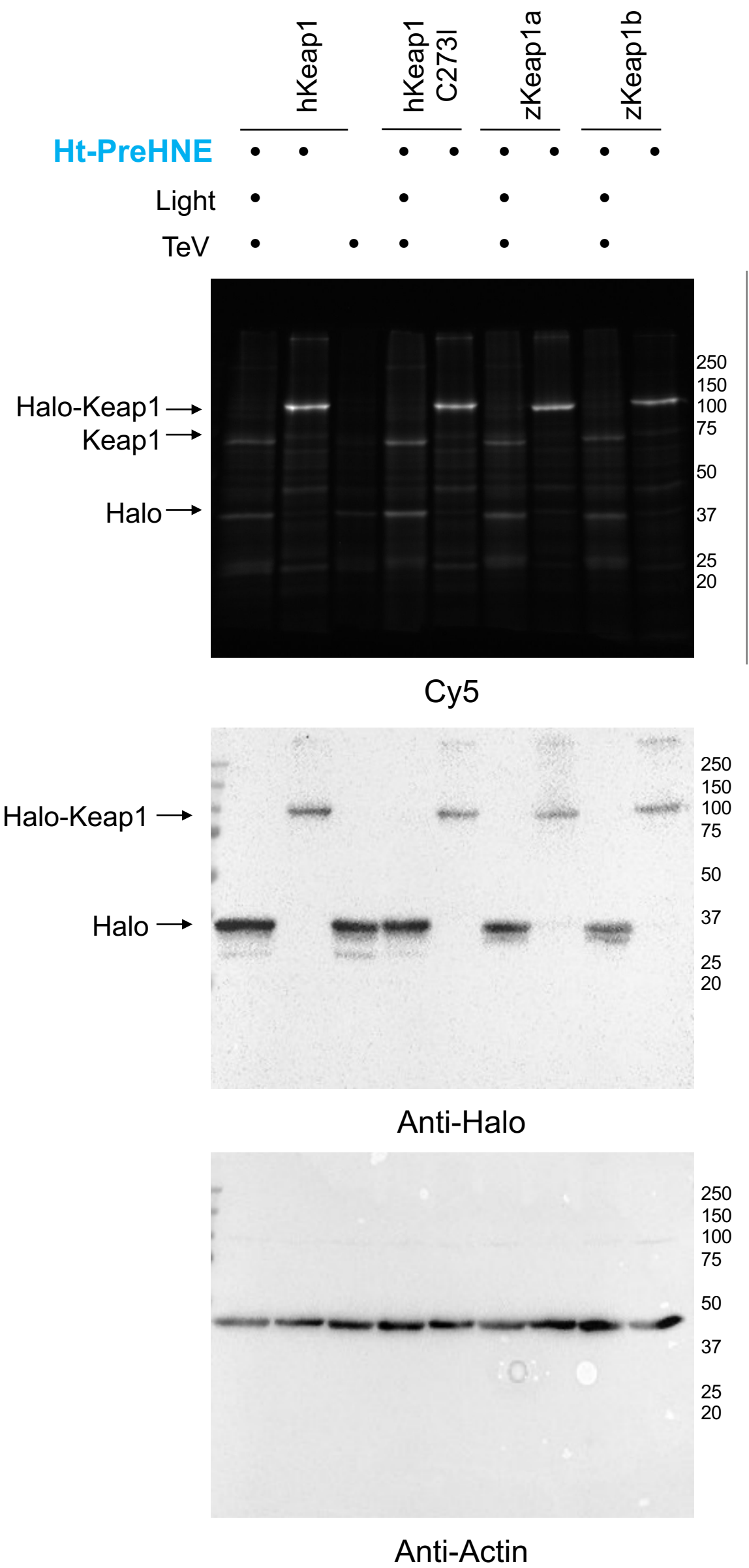

C

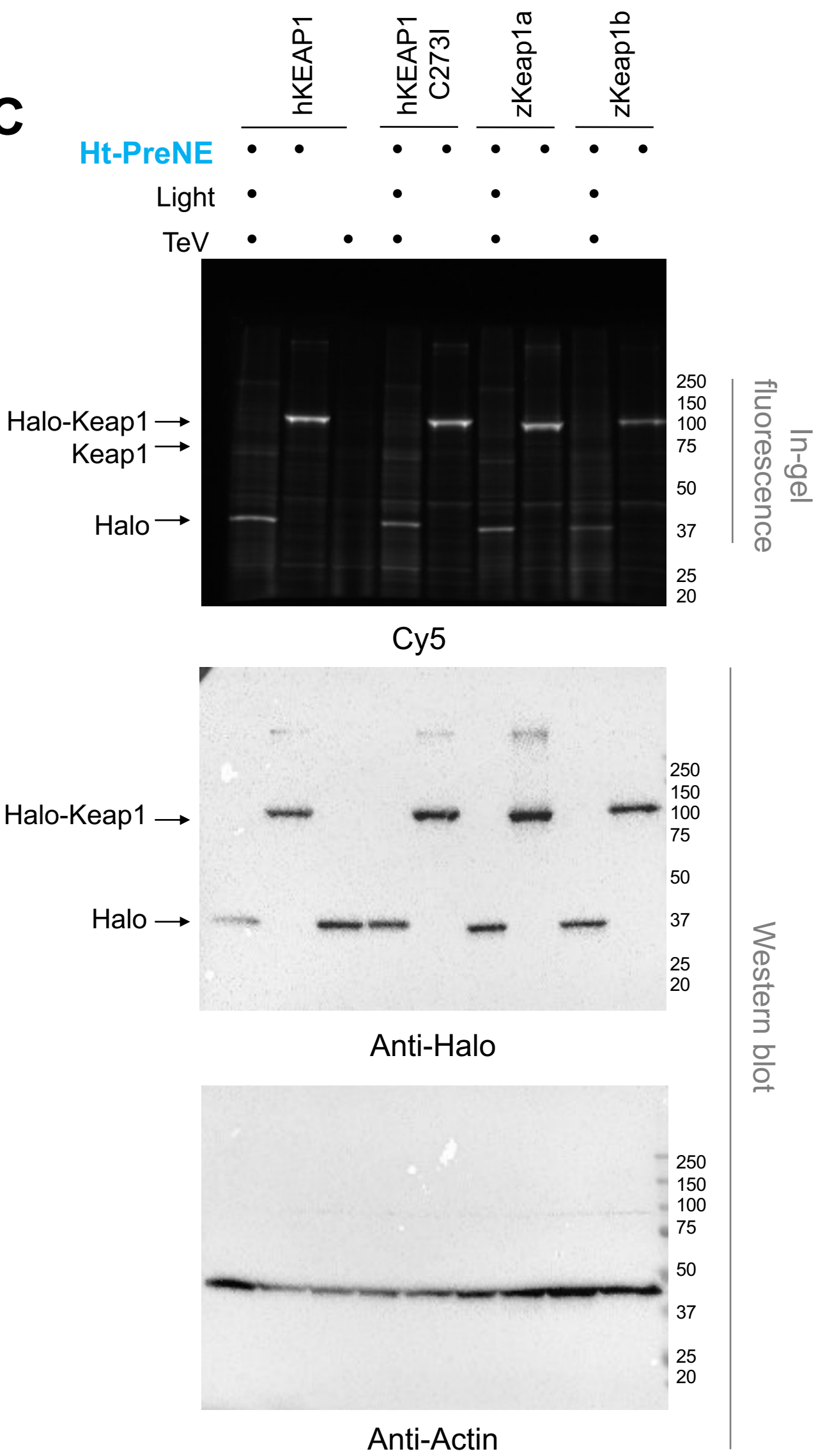

Supplement: Figure 7—figure supplement 2—source data 1. [file elife-83373-fig7-figsupp2-data1.zip › Figure 7-figure supplement 2-source data 1-full view gel and blot image/full view gel and blot.pdf]

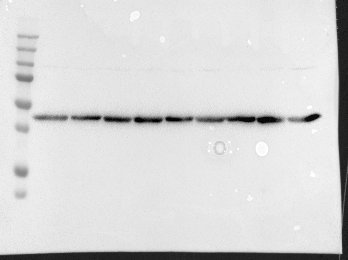

Supplement: Figure 7—figure supplement 2—source data 2. [file elife-83373-fig7-figsupp2-data2.zip › Figure 7-figure supplement 2-source data 2-raw gel and blot image/Figure 7-figure supplement 2b-anti-actin.jpg]

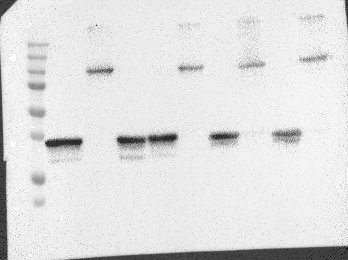

Supplement: Figure 7—figure supplement 2—source data 2. [file elife-83373-fig7-figsupp2-data2.zip › Figure 7-figure supplement 2-source data 2-raw gel and blot image/Figure 7-figure supplement 2b-anti-Halo.jpg]

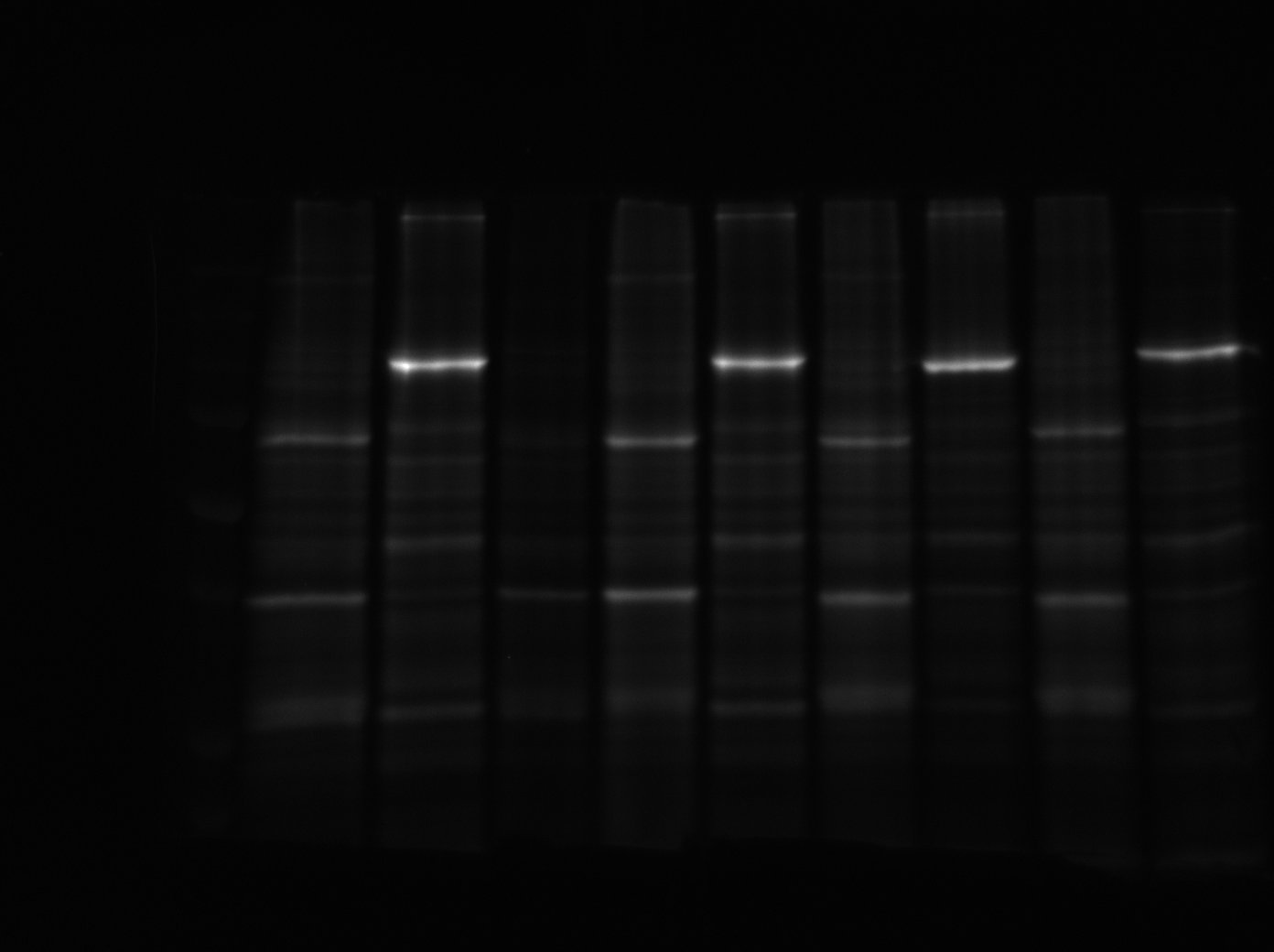

Supplement: Figure 7—figure supplement 2—source data 2. [file elife-83373-fig7-figsupp2-data2.zip › Figure 7-figure supplement 2-source data 2-raw gel and blot image/Figure 7-figure supplement 2b-Cy5.jpg]

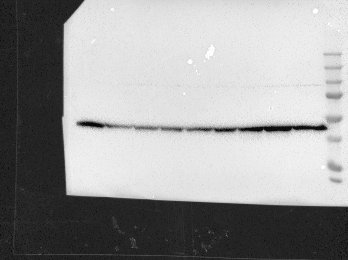

Supplement: Figure 7—figure supplement 2—source data 2. [file elife-83373-fig7-figsupp2-data2.zip › Figure 7-figure supplement 2-source data 2-raw gel and blot image/Figure 7-figure supplement 2c-anti-actin.jpg]

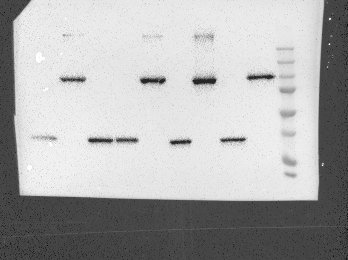

Supplement: Figure 7—figure supplement 2—source data 2. [file elife-83373-fig7-figsupp2-data2.zip › Figure 7-figure supplement 2-source data 2-raw gel and blot image/Figure 7-figure supplement 2c-anti-Halo.jpg]

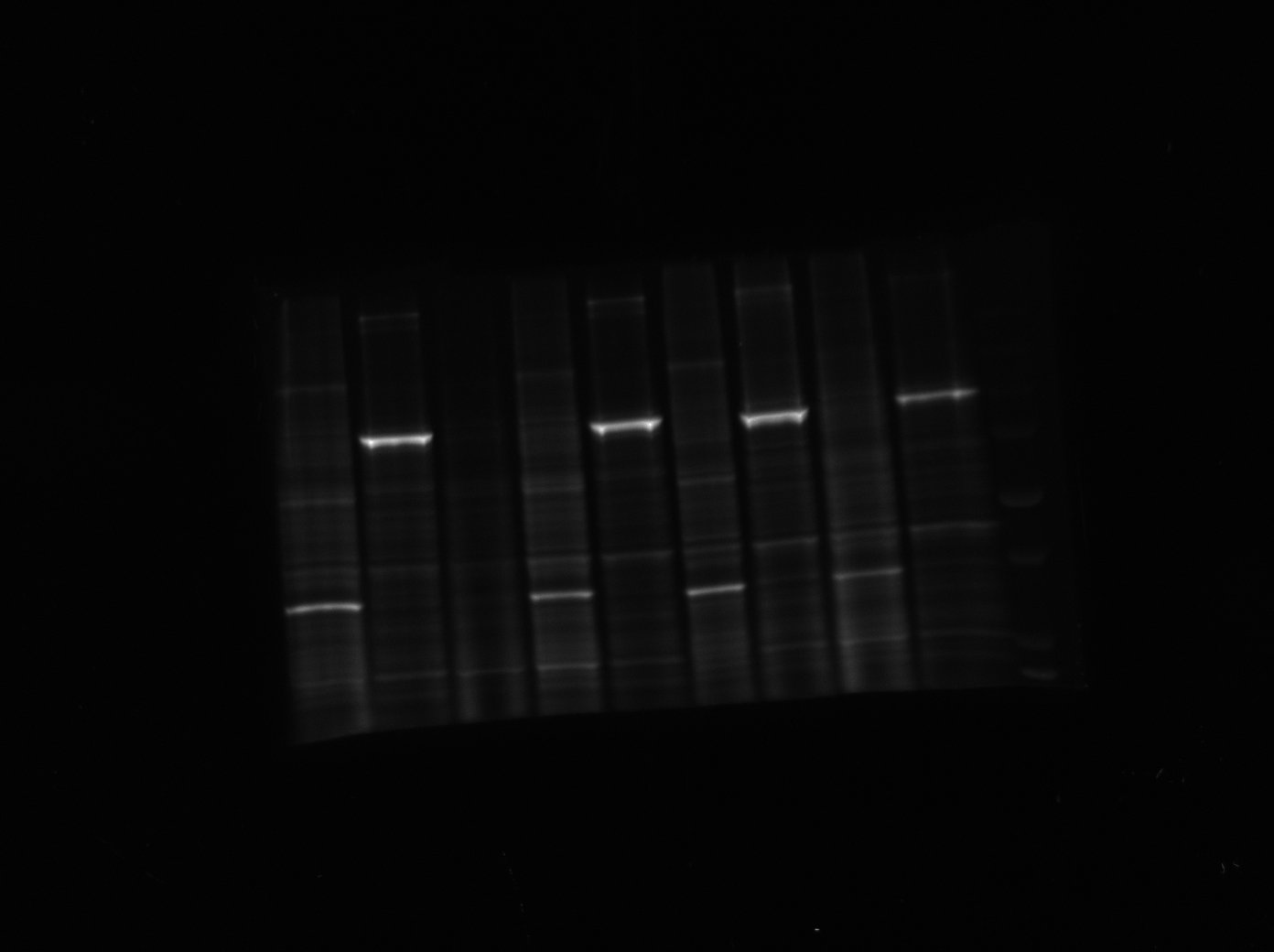

Supplement: Figure 7—figure supplement 2—source data 2. [file elife-83373-fig7-figsupp2-data2.zip › Figure 7-figure supplement 2-source data 2-raw gel and blot image/Figure 7-figure supplement 2c-Cy5.jpg]

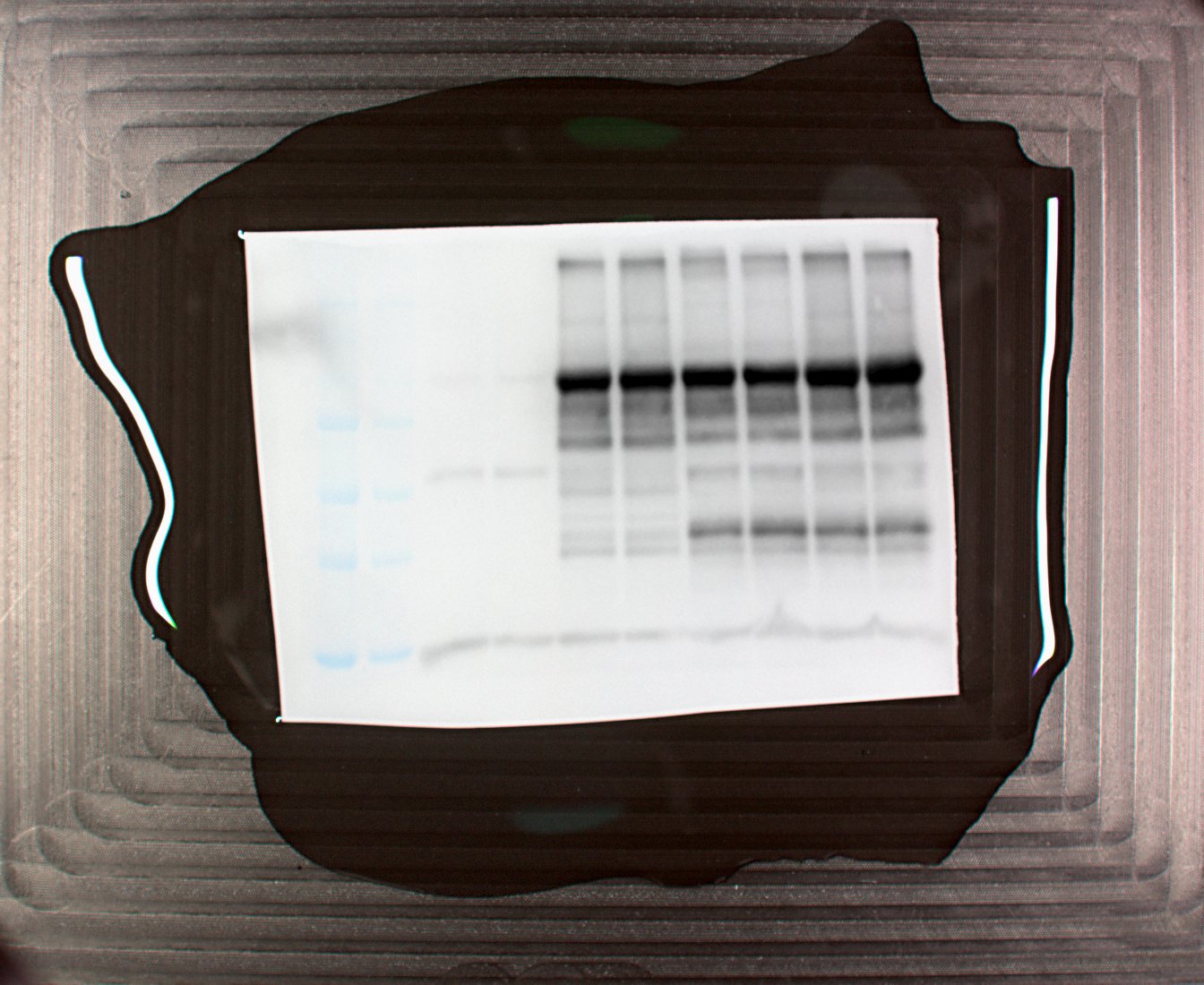

Supplement: Figure 8—source data 2. [file elife-83373-fig8-data2.zip › Figure 8-source data 2-raw blot image/Figure 8a-anti-Flag.jpg]

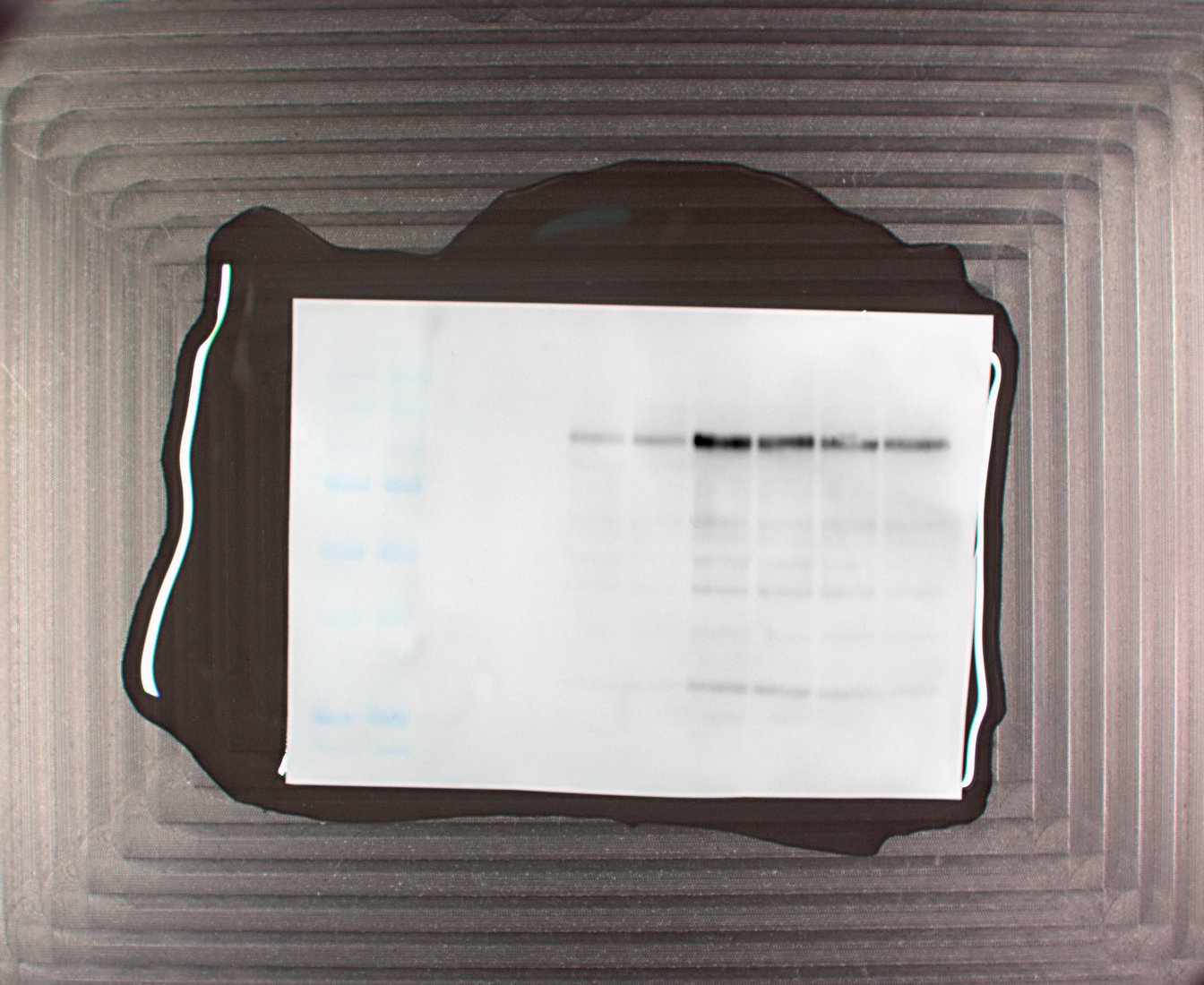

Supplement: Figure 8—source data 2. [file elife-83373-fig8-data2.zip › Figure 8-source data 2-raw blot image/Figure 8a-anti-HA.jpg]

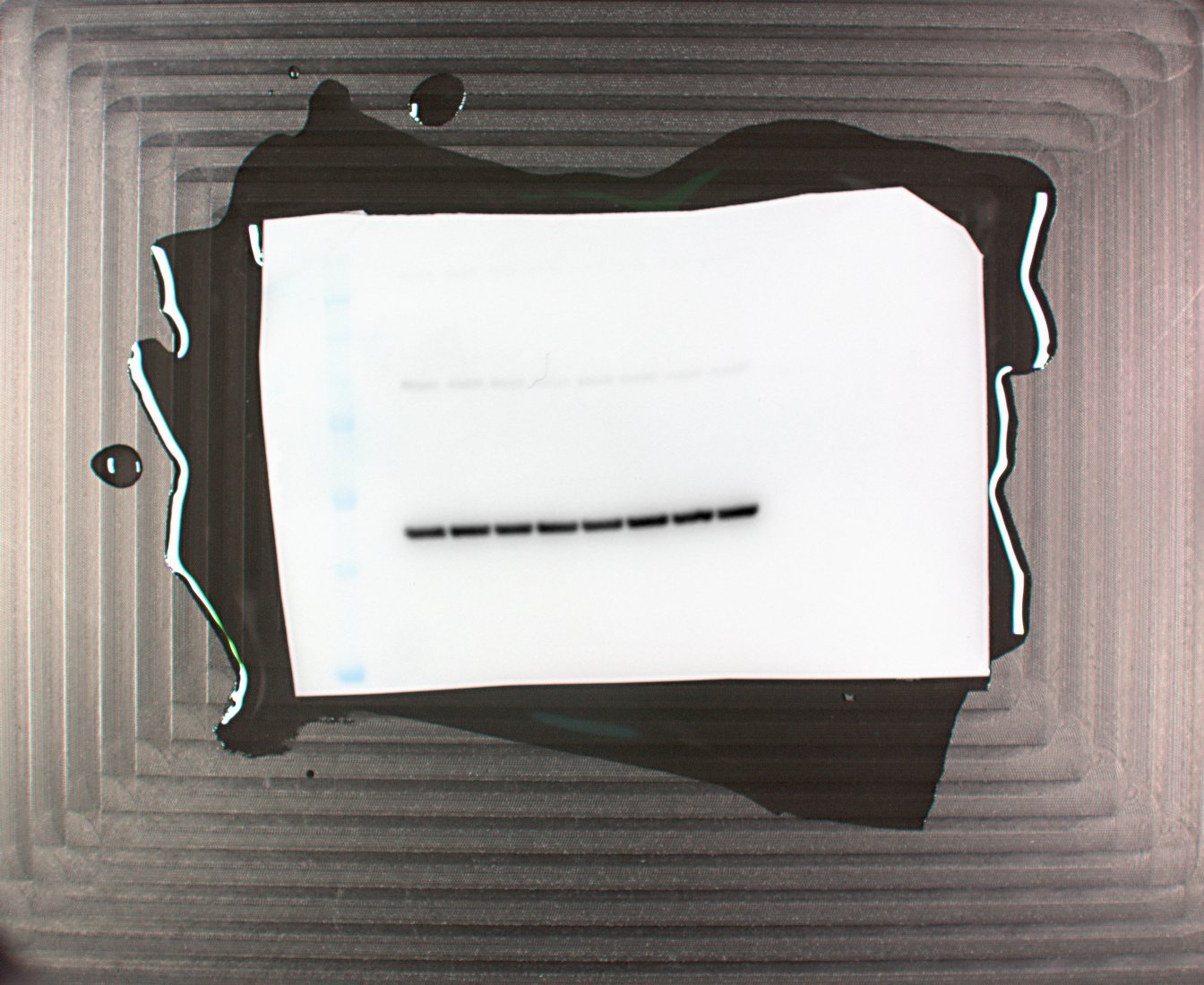

Supplement: Figure 8—figure supplement 1—source data 2. [file elife-83373-fig8-figsupp1-data2.zip › Figure 8-figure supplement 1-source data 2-raw blot image/Figure 8-figure supplement 1-anti-actin.jpg]

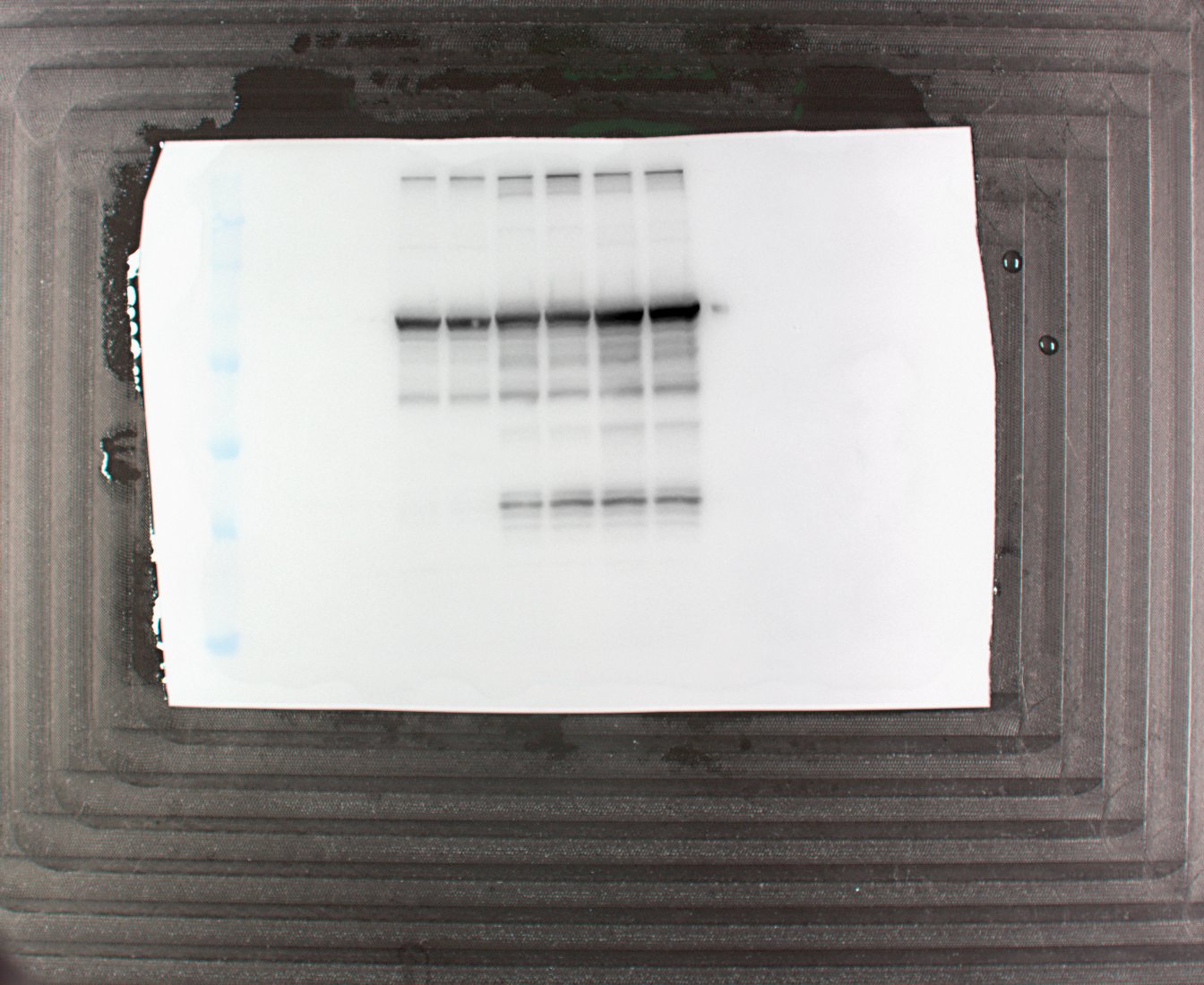

Supplement: Figure 8—figure supplement 1—source data 2. [file elife-83373-fig8-figsupp1-data2.zip › Figure 8-figure supplement 1-source data 2-raw blot image/Figure 8-figure supplement 1-anti-Flag.jpg]

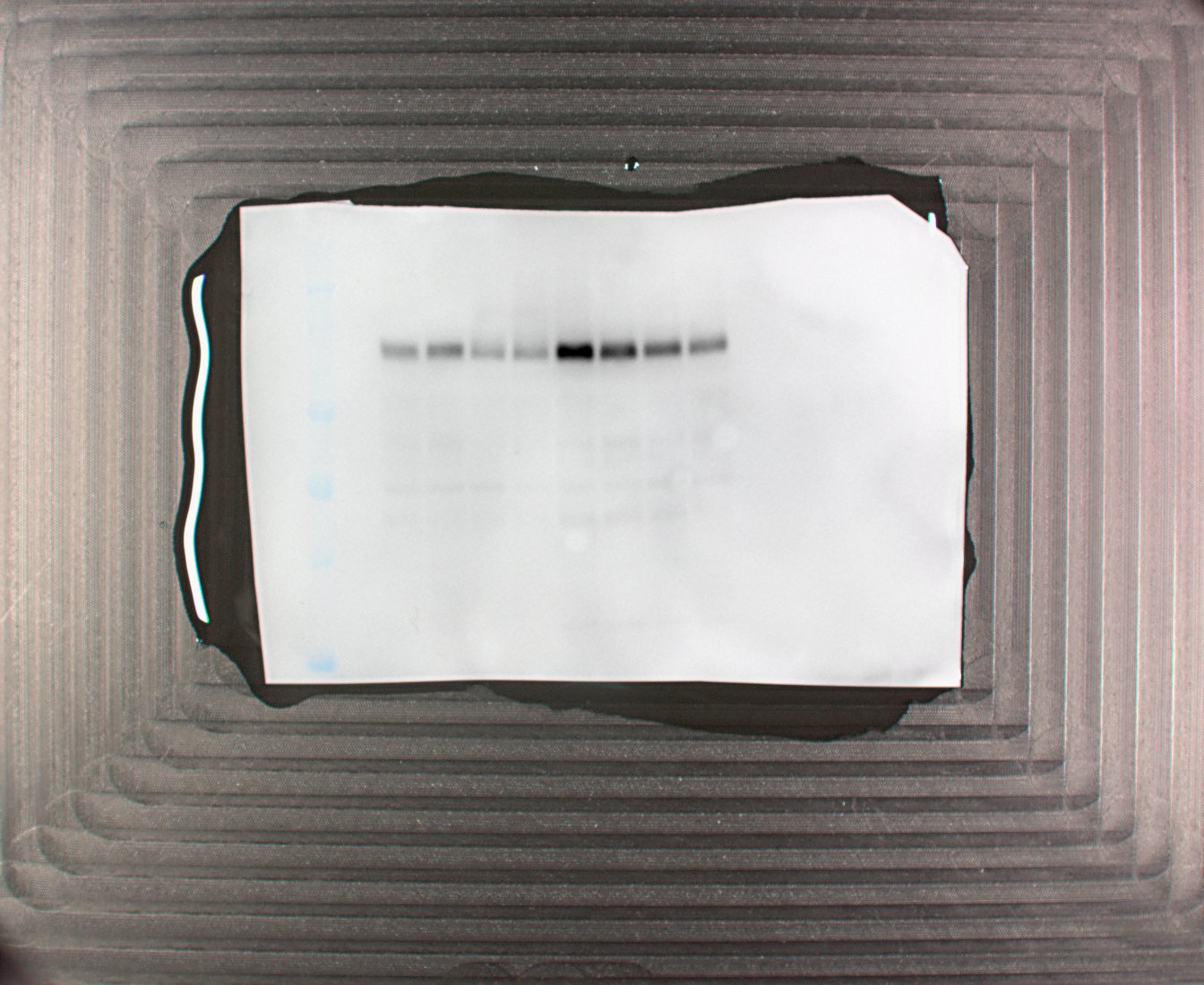

Supplement: Figure 8—figure supplement 1—source data 2. [file elife-83373-fig8-figsupp1-data2.zip › Figure 8-figure supplement 1-source data 2-raw blot image/Figure 8-figure supplement 1-anti-HA.jpg]
