# Supplementary material for: Z-REX uncovers a bifurcation in function of Keap1 paralogs
Source: eLife. 2022 Oct 27;11:e83373. doi: 10.7554/eLife.83373 (PMC9754640; doi:10.7554/eLife.83373)
Supplement: Figure 8—source data 1. [file elife-83373-fig8-data1.zip › Figure 8-source data 1-full view blot image/full view blot.pdf]

Figure 8

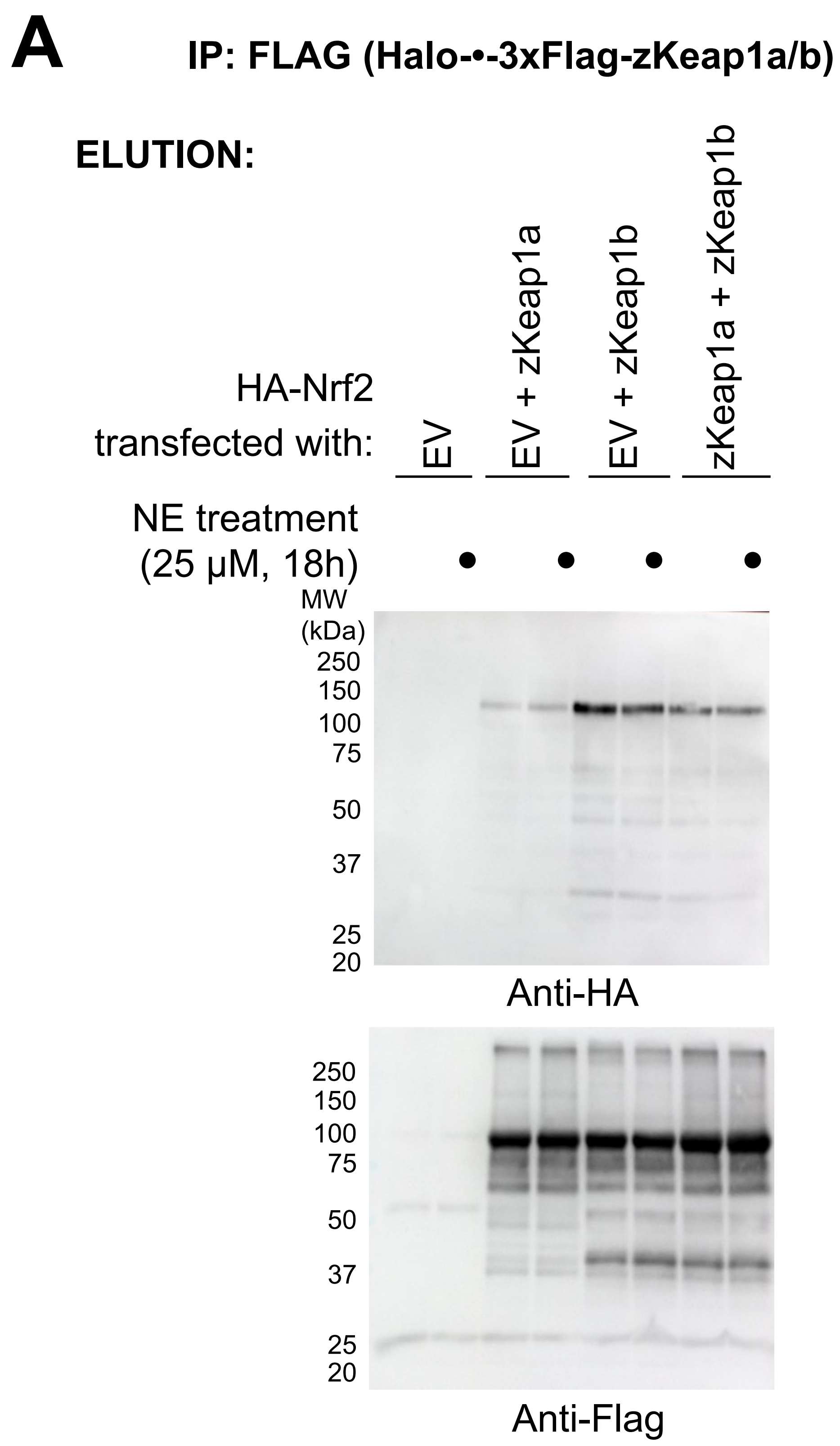
A
IP: FLAG (Halo-•-3xFlag-zKeap1a/b)
ELUTION:
HA-Nrf2
transfected with:
EV
EV + zKeap1a
EV + zKeap1b
zKeap1a + zKeap1b
NE treatment
(25 µM, 18h)
MW
(kDa)
250
150
100
75
50
37
25
20
Anti-HA
250
150
100
75
50
37
25
20
Anti-Flag
