# Supplementary material for: Z-REX uncovers a bifurcation in function of Keap1 paralogs
Source: eLife. 2022 Oct 27;11:e83373. doi: 10.7554/eLife.83373 (PMC9754640; doi:10.7554/eLife.83373)

Figure 8—figure supplement 1

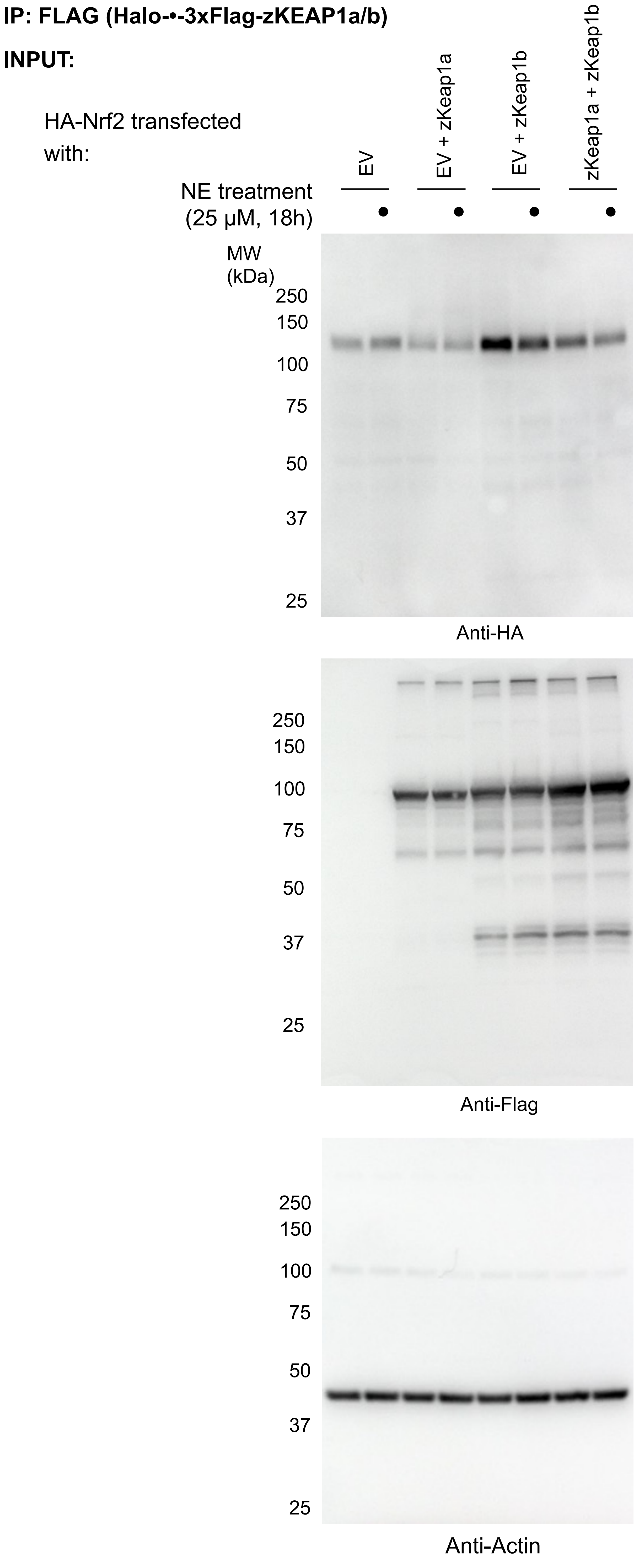

Supplement: Figure 8—figure supplement 1—source data 1. [file elife-83373-fig8-figsupp1-data1.zip › Figure 8-figure supplement 1-source data 1-full view blot image/full view blot.pdf]
