# Supplementary material for: Z-REX uncovers a bifurcation in function of Keap1 paralogs
Source: eLife. 2022 Oct 27;11:e83373. doi: 10.7554/eLife.83373 (PMC9754640; doi:10.7554/eLife.83373)
Supplement: MDAR checklist [file elife-83373-mdarchecklist1.pdf]

eLife

Westbrook Centre
Milton Road
Cambridge CB4 1YG, UK

P 01223 855340
W elifesciences.org
T @elife

Materials Design Analysis Reporting (MDAR) Checklist for Authors

The MDAR framework establishes a minimum set of requirements in transparent reporting mainly applicable to studies in the life sciences.

eLife asks authors to **provide detailed information within their article** to facilitate the interpretation and replication of their work. Authors can also upload supporting materials to comply with relevant reporting guidelines for health-related research (see EQUATOR Network), life science research (see the BioSharing Information Resource), or animal research (see the ARRIVE Guidelines and the STRANGE Framework; for details, see *eLife*'s Journal Policies). Where applicable, authors should refer to any relevant reporting standards materials in this form.

For all that apply, please note **where in the article** the information is provided. Please note that we also collect information about data availability and ethics in the submission form.

Materials:

Newly created materials	Indicate where provided: section/figure legend	N/A
The manuscript includes a dedicated "materials availability statement" providing transparent disclosure about availability of newly created materials including details on how materials can be accessed and describing any restrictions on access.	Page 38, Main manuscript	

Antibodies	Indicate where provided: section/figure legend	N/A
For commercial reagents, provide supplier name, catalogue number and RRID, if available.	Page 24, Key Resources Table	

DNA and RNA sequences	Indicate where provided: section/figure legend	N/A
Short novel DNA or RNA including primers, probes: Sequences should be included or deposited in a public repository.	Primers for cloning; Primers for in vitro transcription; Sequence identity of morpholinos; Primers for morpholino validations; and Primers for qRT-PCR are reported in Appendix	

Cell materials	Indicate where provided: section/figure legend	N/A
Cell lines: Provide species information, strain. Provide accession number in repository OR supplier name, catalog number, clone number, OR RRID.	Page 24 (Main Manuscript: key resources table)	
Primary cultures: Provide species, strain, sex of origin, genetic modification status.	Non-applicable	

Experimental animals	Indicate where provided: section/figure legend	N/A
Laboratory animals or Model organisms: Provide species, strain, sex, age, genetic modification status. Provide accession number in repository OR supplier name, catalog number, clone number, OR RRID.	Page 24 (Main Manuscript: key resources table). NOTE: strain and genetic modification status are available in the original repository websites (URLs provided in the table). Please see page 26 for declarations regarding animal sex. Age of the animals is reported in corresponding figure legends. Briefly, Age: Typically, 32–36 hours post-fertilization (hpf); unless otherwise indicated. Sex: It is not possible to sex D. rerio at the developmental stage we used. However, it is likely that the pool of embryos in each experiment contained roughly equal numbers of males and females.	
Animal observed in or captured from the field: Provide species, sex, and age where possible.	Non-applicable	

Plants and microbes	Indicate where provided: section/figure legend	N/A
Plants: provide species and strain, ecotype and cultivar where relevant, unique accession number if available, and source (including location for collected wild specimens).	Non-applicable	
Microbes: provide species and strain, unique accession number if available, and source.	Non-applicable	

Human research participants	Indicate where provided: section/figure legend) or state if these demographics were not collected	N/A
If collected and within the bounds of privacy constraints report on age, sex, gender and ethnicity for all study participants.	Non-applicable	

Design:

Study protocol	Indicate where provided: section/figure legend	N/A
If the study protocol has been pre-registered, provide DOI. For clinical trials, provide the trial registration number OR cite DOI.	Non-applicable	

Laboratory protocol	Indicate where provided: section/figure legend	N/A
Provide DOI OR other citation details if detailed step-by-step protocols are available.	All step-by-step protocols are reported in detail in Methods & Materials section of the Main Manuscript	

Experimental study design (statistics details) *		
For in vivo studies: State whether and how the following have been done	Indicate where provided: section/figure legend. If it could have been done, but was not, write "not done"	N/A
Sample size determination	As indicated in Statistical Analysis & Reporting section (Main Manuscript, page 26), no statistical methods were used to pre-determine sample size. Size of datasets was chosen according to literature and based on our own experience, integrating similar methods of analysis. Number of technical replicates and biological replicates are reported in figure legends.	
Randomisation	As indicated in Statistical Analysis & Reporting section (Main Manuscript, page 26), for zebrafish experiments, all injected embryos for a given injection condition were pooled and mixed prior to being divided equally into experimental groups without bias. Cell-based experiments were set up with cells derived from a common stock and divided equally without bias. Treatment groups for all experiments were assigned randomly.	
Blinding	Blinding was not used in this study, consistent with widespread practice in the field for studies of this nature. Nevertheless, we ensured rigor by having multiple co-authors reproduce the discovered phenotypes independently.	
Inclusion/exclusion criteria	No data were excluded.	

Sample definition and in-laboratory replication	Indicate where provided: section/figure legend	N/A

State number of times the experiment was replicated in the laboratory.	Indicated in corresponding figure legends. Briefly, all experiments were conducted with sufficient biological replicates to ensure rigor. Generally, the pathways / mode-of-mechanisms we identified were confirmed with orthogonal approaches (e.g., genetic knockdown/knockout and pharmacological modulators of key proteins), and pathway functionality was confirmed/replicated in multiple model systems (zebrafish and cultured mammalian cells). Additionally, identified phenotypes were confirmed in multiple zebrafish strains. We list the number of independent biological vs. technical replicates for each experiment in the corresponding figure legends	
Define whether data describe technical or biological replicates.	No. of independent biological vs. technical replicates for each experiment is indicated in the corresponding figure legends	

Ethics	Indicate where provided: section/submission form	N/A
Studies involving human participants: State details of authority granting ethics approval (IRB or equivalent committee(s), provide reference number for approval.	Non-applicable	
Studies involving experimental animals: State details of authority granting ethics approval (IRB or equivalent committee(s), provide reference number for approval.	All procedures performed at Cornell (2017-2018) and EPFL (2018-present) conform to the animal care, maintenance, and experimentation procedures followed by Cornell University's and EPFL's Institutional Animal Care and Use Committee (IACUC) guidelines and approved by the respective institutional committees. All experiments with zebrafish performed at EPFL (2018-present) have been performed in accordance with the Swiss regulations on Animal Experimentation (Animal Welfare Act SR 455 and Animal Welfare Ordinance SR 455.1), in the EPFL zebrafish unit, cantonal veterinary authorization VD-H23).	
Studies involving specimen and field samples: State if relevant permits obtained, provide details of authority approving study; if none were required, explain why.	Non-applicable	

Dual Use Research of Concern (DURC)	Indicate where provided: section/submission form	N/A
If study is subject to dual use research of concern regulations, state the authority granting approval and reference number for the regulatory approval.	Non-applicable	

Analysis:

Attrition	Indicate where provided: section/figure legend	N/A
Describe whether exclusion criteria were pre-established. Report if sample or data points were omitted from analysis. If yes, report if this was due to attrition or intentional exclusion and provide justification.	No data were excluded. Outliers were maintained in all data sets with error bars designating SEM and P-values from application of two-tailed Students' t-test included, as indicated in corresponding figure legends	

Statistics	Indicate where provided: section/figure legend	N/A
Describe statistical tests used and justify choice of tests.	Described in the 'Statistical analysis & reporting section' in Main Manuscript (Page 25), and associated figure legends	

Data availability	Indicate where provided: section/submission form	N/A
For newly created and reused datasets, the manuscript includes a data availability statement that provides details for access (or notes restrictions on access).	Page 38, Main Manuscript	
When newly created datasets are publicly available, provide accession number in repository OR DOI and licensing details where available.	Non-applicable	
If reused data is publicly available provide accession number in repository OR DOI, OR URL, OR citation.	Non-applicable	

Code availability	Indicate where provided: section/figure legend	N/A
For any computer code/software/mathematical algorithms essential for replicating the main findings of the study, whether newly generated or re-used, the manuscript includes a data availability statement that provides details for access or notes restrictions.	Non-applicable	
Where newly generated code is publicly available, provide accession number in repository, OR DOI OR URL and licensing details where available. State any restrictions on code availability or accessibility.	Non-applicable	

If reused code is publicly available provide accession number in repository OR DOI OR URL, OR citation.	Non-applicable	

Reporting:

The MDAR framework recommends adoption of discipline-specific guidelines, established and endorsed through community initiatives.

Adherence to community standards	Indicate where provided: section/figure legend	N/A
State if relevant guidelines (e.g., ICMJE, MIBBI, ARRIVE, STRANGE) have been followed, and whether a checklist (e.g., CONSORT, PRISMA, ARRIVE) is provided with the manuscript.	As described above and also briefly stated in the 'Statistical analysis & reporting section' in Main Manuscript (Page 26), our reporting protocol of experimental procedures, data analyses and interpretations are consistent with the 'ARRIVE essential 10' guidelines (as outlined in, for instance, Percie du Sert et al., 2020 PLOS Biology)	

---

* We provide the following guidance regarding transparent reporting and statistics; we also refer authors to Ten common statistical mistakes to watch out for when writing or reviewing a manuscript.

Sample-size estimation

- You should state whether an appropriate sample size was computed when the study was being designed
- You should state the statistical method of sample size computation and any required assumptions
- If no explicit power analysis was used, you should describe how you decided what sample (replicate) size (number) to use

Figure legends contain information pertaining to the following:

- the exact sample size (n) for each experimental group/condition, given as a discrete number and unit of measurement;
- --about whether measurements were taken from distinct samples or whether the same sample was measured repeatedly;
- -- null hypothesis testing (two-tailed Students' t-test) with exact P values noted whenever suitable.
- No statistical methods were used to pre-determine sample size. Size of datasets was chosen according to literature and based on our own experience, integrating similar methods of analysis. Number of technical replicates and biological replicates are reported in figure legends.

Replicates

- You should report how often each experiment was performed
- You should include a definition of biological versus technical replication
- The data obtained should be provided and sufficient information should be provided to indicate the number of independent biological and/or technical replicates
- If you encountered any outliers, you should describe how these were handled
- Criteria for exclusion/inclusion of data should be clearly stated
- High-throughput sequence data should be uploaded before submission, with a private link for reviewers provided (these are available from both GEO and ArrayExpress)

- Figure legends contain description of independent biological replicates, vs. technical replicates.
- Outliers were maintained in all data sets with error bars designating SEM and P-values from application of two-tailed Students' t-test included.

Statistical reporting

- Statistical analysis methods should be described and justified
- Raw data should be presented in figures whenever informative to do so (typically when N per group is less than 10)
- For each experiment, you should identify the statistical tests used, exact values of N, definitions of center, methods of multiple test correction, and dispersion and precision measures (e.g., mean, median, SD, SEM, confidence intervals; and, for the major substantive results, a measure of effect size (e.g., Pearson's r, Cohen's d)
- Report exact p-values wherever possible alongside the summary statistics and 95% confidence intervals. These should be reported for all key questions and not only when the p-value is less than 0.05.

- Wherever applicable, figure legends contain information pertaining to SEM with associated P values, sample size (e.g., number of fish embryos analyzed, number of independent biological replicates). Representative raw images are included with accompanying quantitation where relevant.

Group allocation

- Indicate how samples were allocated into experimental groups (in the case of clinical studies, please specify allocation to treatment method); if randomization was used, please also state if restricted randomization was applied
- Indicate if masking was used during group allocation, data collection and/or data analysis

- Summary information related to sample allocation/handling is detailed in the supplementary text.
- Briefly, no masking was used. Prior to beginning each experiment, cells/embryos were allocated into groups randomly, for each sample group. When an experiment was commenced, groups of cells/embryos were allocated into treatment groups without pattern or bias. This ensured that each treatment group in an experiment was identical to account for any variation across cells/fish breeding. Cell counting was performed at each step of the experiment whenever relevant, to rigorously standardize conditions both within each experiment and across different experiments. All procedures related to zebrafish studies conform to the respective NIH and European guidelines regarding animal experimentation and were approved by the corresponding Institutional Animal Care and Use committees at EPFL (Switzerland) and Cornell University (USA) (the authors' former institution where the initial work on this manuscript was performed). Casper strain zebrafish, wild-type zebrafish, and previously-validated reporter strains were used for the experiments involving embryos and for the latter transgenic reporter strains, at consistent zygosity. As zebrafish are believed to exhibit polygenic sex determination, at the age at which experiments were undertaken, the sex of the fish was unable to be determined, but likely account for 50:50 male: female.

Additional data files ("source data")

Supporting information figures and Source Data file contains all of the requisite data (both representative raw images and corresponding quantitation). There are no large-scale data sets (such as transcriptomic-sequencing or proteomics data) associated with this manuscript.
